# Structure, tribotechnical, and thermophysical characteristics of the fluoroplastic carbonnanotubes material

**DOI:** 10.1186/1556-276X-9-213

**Published:** 2014-05-06

**Authors:** Sergiy Revo, Alexandre Alekseev, Ekaterina Ivanenko, Toufik Labii, Abdelhamid Boubertakh, Smail Hamamda

**Affiliations:** 1 Taras Shevchenko National University of Kyiv, 64/13, Volodymyrska Street, 01601 Kyiv, Ukraine; 2 Laboratory of Thermodynamics and Surface Treatment of Materials University Constantine1, B.P. 325 Route Ain El Bey, Constantine 25017, Algeria

**Keywords:** Nanocomposite, Friction coefficient, Wear resistance, Thermal expansion, Anisotropy

## Abstract

In this work, we studied a nanocomposite material made from fluoroplastic which contains 20 wt.% multi-walled nanotubes. In order to complete the present work, we have used different thermodynamic and mechanical techniques. The introduction of nanotubes in the F4 polymer matrix has completely changed the tribological and thermodynamic properties of the studied nanocomposite material. The compression strength becomes 20% higher than that of the F4 polymer matrix. Meanwhile the wear resistance achieves an order of magnitude 100 times greaterthan that of F4. Moreover, a friction coefficient is about 25% to 30% lower than that of a similar material and especially that of F4 material. Differential scanning calorimetric study showed that the glassy phase transition appears at about 330°C, which confirms that the degradation of the studied nanocomposite occurs at relatively higher temperature. This result confirms the one concerning the change in tribological properties. Dilatometric study revealed that the thermal expansion coefficient has been increased. The observed relative elongation measurement change depends on the direction along which the measurement has been done and confirms, in turn, the anisotropic character of the studied material. These results suggest that the metallic materials could be replaced by nanocomposite compounds which present good physical properties.

## Review

### Introduction

The emphasis for nanocomposite materials by the scientific community and the industry continues to grow and to develop. The new allotropes of carbon transformations observed recently give to this material a privileged place and as well as an interesting prospect in various fields such as energy, mechanics, and superconductivity [1-6].

The high performance of polymer nanocomposites offers new perspectives in the materials science field. The substitution of heavy metal parts in many applications has become possible, thanks to the benefits offered by polymers containing carbon nanotubes. Lightness, elasticity, and corrosion resistance make these nanocomposites very competitive in various fields of technology [7-9].

The intensification of industrial processes today is to greatly extend based on the durability of machine assembly units and equipment working in friction units. This durability is of particular importance for friction units which operate in extreme conditions, particularly in a hostile environment, at high temperatures, etc. Thus, there is the need of development of new wear-resistant materials with a low friction coefficient (k_fr_), high values of wear resistance with thermal conductivity, which would be resistant to hostile environments. The latter is a topical issue in our days, although there is no unique solution to the cited above issue. Indeed, there are several ways to extend the capability of the existing materials in order to be used in the abovementioned conditions.

### Experimental

In the present study, we investigate the possibility of making a new wear-resistant material in hostile environments, the nanocomposite materials (NCM) based on a fluoroplastic matrix F4 and on multi-walled carbon nanotubes (MCNT). These nanotubes were obtained by CVD method in a rotating reactor [10]. Al_2_O_3_-MoO_3_-Fe_2_O_3_ types were used as catalyst. Propylene was used as a source of carbon. The fluoroplastic water suspension was thoroughly mixed with deagglomerated and dried MCNT. The mixture was pressed at a temperature *T* = 350°С ± 0.5°С and under a pressure *Р* = 500 MPa. The structure of the samples was studied using an optical microscope (Neofot type) and a scanning electron microscope, their tribotechnical characteristics by a laboratory instrument of UMT-1 type, and their thermophysical characteristics by SETARAM DSC 92 instrument (Grand Prairie, TX, USA) and DIL 402C NETZSCH dilatometer (NETZSCH, Annaba, Algeria). For dilatometric investigations, the radial (*R*) and axial (*Z*) directions to the sample pressing were considered. The *α*(*T*) measurements were made with a precision of about 10^-7^°C^-1^. The relative error in determining *k*_fr_ did not exceed 4%; in determining the degree of wear by the decrease of mass due to friction against the counterface (Cr-W-Mn steel) with no lubricant, the relative error did not exceed 7%. The speed of sliding friction was selected in the range of 1.25 to 10 m/s, with the load on the samples of 0.4 to 1.1 MPa. The degree of wear was determined within the sliding distance of 1,000 m.

### Results and discussion

**Figure 1:**
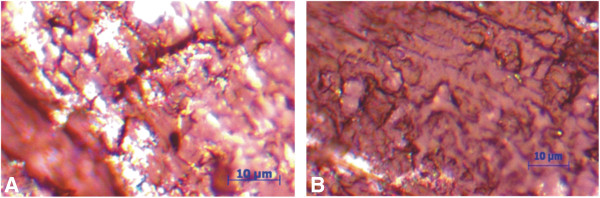
Microstructure of the fluoroplastic nonagglomerated MCNT nanocomposite material (A) and the fluoroplastic deagglomerated MCNT nanocomposite material (B).

Both degrees of tribotechnical and thermophysical characteristics of NCM depend on several factors, while its thermal conductivity and the heat abstraction rate from the friction area are, for the most part, responsible for the wear resistance in a friction pair. This is particularly true for polymer compositions. An important factor in this case is the uniformity of a filler distribution in the NCM matrix, as one can see from the NCM structure shown in Figure 1. The applied method for the samples’ production has provided more or less a uniform MCNT distribution in the fluoroplastic matrix. In turn, this provides, for a low percolation, a threshold for the composition: according to the data on the concentration dependence of the electrical resistance, which is of the order of *С*_*С*_ = (4.1 ± 0.1) vol.% of MCNT. The density of the obtained NCM samples remains the same as that of F4, which is about 2.1 to 2.2 g/cm^3^ at room temperature. The maximum compression strength was obtained for the NCM with the MCNT concentration of 20 wt.% and its value is *σ*_compr_ = 55 ± 3 MPa, which is 20% higher than that of the F4. The elastic modulus, which is of particular importance, and the yield point for NCM samples are also higher compared to the respective values of the matrix obtained in the same way. The friction coefficient at a speed of 5 m/s decreases for the industrial fluoroplastic from 0.14 to 0.05 on increasing the applied load to the samples from 1 to 20 kg/cm^2^, whereas it decreases in the same case between 25% and 30% for our NCM samples, with a lubricant coefficient, *k*_fr_, which decreases two times compared to that of the matrix. The substantial advantage of our developed material is that its wear resistance compared to similar materials and, especially, to F4 is 100 times higher.

**Figure 2:**
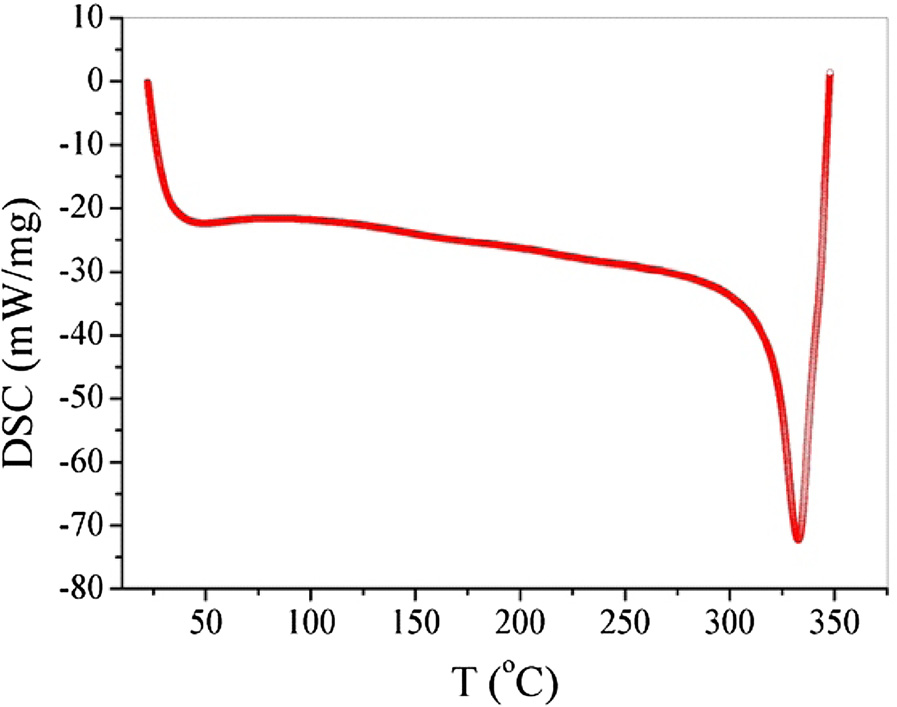
Differential scanning calorimetric diagram of fluoroplastic MCNT nanocomposite materials obtained with a heating rate of 10°C/min.

It is important to note that according to the results of thermal conductivity studies and those of differential scanning calorimetry (DSC), it can be stated that no destruction of the NCM’s matrix is observed during heating treatments up to a temperature of 330°С, Figure 2. Indeed at this temperature, we observe a heat release peak of the studied samples. The nanotubes introduction has shift the transition temperature of the glassy phase towards higher temperatures [11,12].

**Figure 3:**
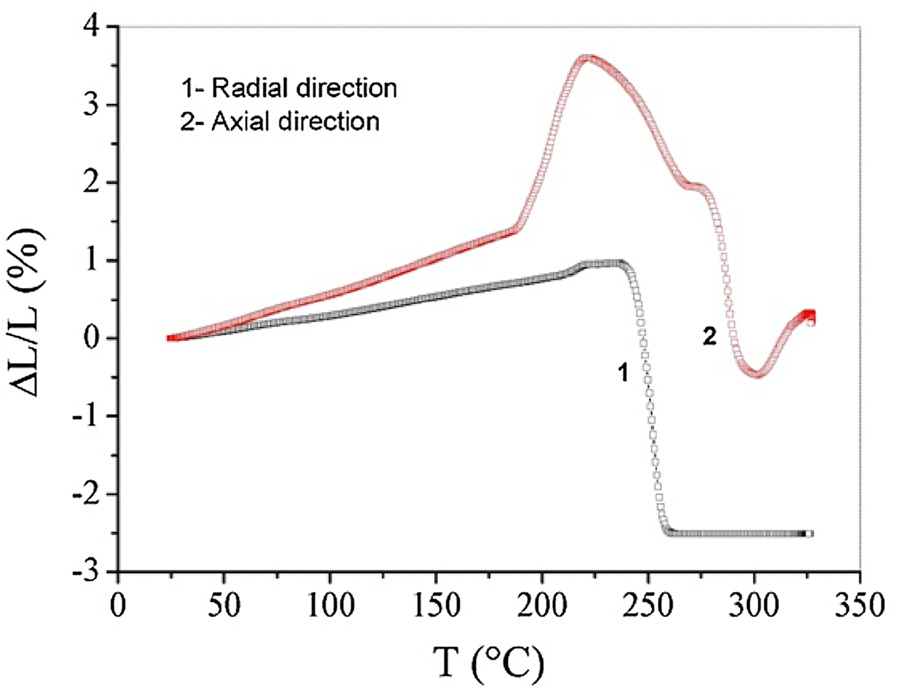
Linear relative elongation of fluoroplastic MCNT nanocomposite material samples at different temperatures (heating rate, 10°C/min).

**Figure 4:**
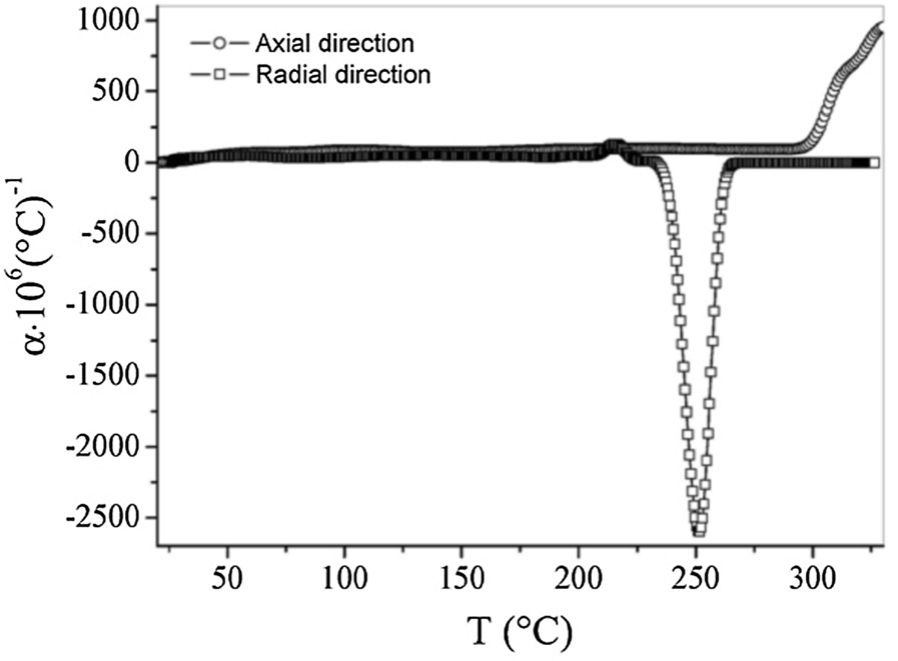
Thermal expansion coefficient of fluoroplastic MCNT nanocomposite material samples as a function of temperature (heating rate, 10°C/min).

The study of the temperature dependence of the linear thermal expansion coefficient, *α*(*T*), and the samples’ relative elongation Δ*L*/*L* enabled us to find out the characteristics of the dependence of *α*(*T*) and Δ*L*/*L* upon the temperature (Figures 3 and 4). Figure 3 showed the nature of the studied anisotropic nanocomposite. The curves show the relative elongation changes of the sample and reveal the presence of anomalies whose shapes and intensities vary from the axial direction to the radial one.

The data provided here is the evidence of devitrification of areas of the polymer matrix, which is accompanied by an increase of the composite’s deformability and an increase of its thermal expansion coefficient. This established effect must be taken into account when selecting a working temperature range for the friction units based on this developed material. Due to the fewer works reported in this domain, it is important to start by a discussion of the obtained dilatometric results.

The thermal expansion behavior of the studied nanomaterial (discs of 39.8 mm in diameter and height of about 4.36 mm) depends strongly on both measuring directions (radial (*R*) and axial (*Z*)). The shape of *α*(*T*) curves depends on the measuring direction. It important to note that the studied material is anisotropic. This result is consistent with those reported by other researchers elsewhere [13].

In the temperature range of 20°C to 170°C, the thermal expansion coefficient as a function of temperature measured along the axial direction *α*_Z_(*T*) (pressing direction) is greater than that obtained from the radial direction *α*_R_(*T*) over all this temperature range. The mean values of the axial and the radial thermal expansion coefficients are positive and equal to 80 and 40 10^-6^°C^-1^, respectively. From 230°C, both of them become negative. Beyond 270°C, α_R_(*T*) becomes positive and goes back to its original value. It is clear that in the regions before and after the anomalous, the *α*_R_(*T*) appears to be constant. While *α*_Z_(*T*) becomes positive from 300°C.

As for dilatometric anomalies, their numbers are also closely linked to the direction of measurement. The *α*_Z_(*T*) curve contains three anomalies, while *α*_R_(*T*) shows only two. The first anomalous in the *α*_Z_(*T*) appearing at around 210°C relatively intense. Its intensity is equal to 1,000 10^-6^°C^-1^. The latter intensity is 10 times greater than that of *α*_R_(*T*) whose intensity is not more than 100 10^-6^°C^-1^ and which appears in delay by 20°C compared the that in the case of *α*_Z_(*T*). For the case of the second anomalous, the roles are reversed. The dilatometric peak of *α*_R_(*T*) appears before *α*_Z_(*T*), and the ratio *α*_R_(*T*)/*α*_Z_(*T*) is about 500%. At 280°C, *α*_Z_(*T*) shows a significant anomaly, which is not observed in the case of the *α*_R_(*T*) curve.

It is important to note that the thermal expansion coefficient values obtained in the present work are of the same order of magnitude as those calculated by other authors [12,14] using the dynamic molecular theory.

## Conclusions

At the end of this study, we can conclude that the studied nanomaterial is of a great interest. It gives the compromise between the results obtained by different techniques. The MCNT obtained by the strengthening of the F4 matrix showed a maximum strain for a concentration of 20 wt.% of multi-walled carbon nanotubes. This strain is 20% higher than that of the matrix alone. The value of Young’s modulus is increased by the same proportion. In addition, the friction coefficient is reduced by 25% to 30%, whereas the lubricant coefficient is reduced by 50% compared to that of the matrix resulting in a wear resistance higher about 100 times.

On the other hand, the dilatometric measurements show clearly the existence of two distinct areas. The first one is in between 25°C and 180°C, which shows that the mean values of *α*(*T*) measured along the axial and the radial directions are 80 and 40 10^-6^°C^-1^, respectively. The second region ranges from 190°C to 310°C, in which *α*(*T*) curves show several dilatometric anomalies with very important intensities and their numbers vary depending on the direction along which the measurement has been carried out. The thermal expansion coefficient of the nanocomposite changes from one direction to another, and the relative elongation Δ*L*/*L* measurements along the radial and the axial directions confirm the anisotropic nature of fluoroplastic material containing 20 wt.% of multi-walled nanotubes (MNTC). The DSC diagram shows an intense peak at around 340°C, which is characteristic of the transition from the glassy phase, and suggests that the deterioration of the material appears at high temperature. The mechanical characteristics of our samples were significantly improved. The latter results were confirmed by dilatometric and calorimetric techniques.

## Competing interests

The authors declare that they have no competing interests.

## Authors’ contributions

SR conceived of the study, and participated in its design and result discussion. AA carried out tribotechnical research and result discussion. EI preparing of the nanocomposite samples, carried out microscopic studies and drafted the first version manuscript. TL carried out experimental research of the nanocomposites thermal expansion and drafted the manuscript. AB carried out experimental research of the nanocomposites thermal capacity and result discussion. SH conceived of the study, participated in its design, and result discussion and coordination. All authors read and approved the final manuscript.
